# Thalamic Tumors in a Pediatric Population: Surgical Outcomes and Utilization of High-Definition Fiber Tractography and the Fiber Tracking Technique

**DOI:** 10.7759/cureus.23611

**Published:** 2022-03-29

**Authors:** Abdulelah A Alluhaybi, Khalid S Altuhaini, Lahbib Soualmi, Fahad Alotaibi, Ayman Al Banyan, Maqsood Ahmad

**Affiliations:** 1 Pediatric Neurosurgery, King Fahad Medical City/National Neuroscience Institute, Riyadh, SAU; 2 Neurophysiology, King Fahad Medical City/National Neuroscience Institute, Riyadh, SAU

**Keywords:** glioma, neurogery, pediatric, brain tumors, fiber tracking, dti, iom, thalamic tumors

## Abstract

Objective: This study aimed to assess the operability of thalamic tumors since they are generally considered to be inoperable and to have poor outcomes. Advancements in neuroimaging, neuronavigational technology, and intraoperative neurophysiological monitoring allow accurate planning and safe resection.

Methods: Clinical data and reports of 10 pediatric patients with thalamic tumors were retrieved retrospectively. All 10 patients underwent surgical intervention. Diffusion tensor tractography (DTI) was used preoperatively to select the safest surgical route. Intraoperative MRI and postoperative MRI were used to evaluate the extent of resection.

Results: There were three gross total resections (GTRs), two subtotal resections (STRs), two partial resections (PRs), and three biopsies. All patients had unilateral thalamic tumors. Different surgical approaches were used according to the relationship with the internal capsule and corticospinal tract and according to the preoperative DTI. Five patients had pilocytic astrocytoma, two had diffuse pediatric-type high-grade glioma, one had ganglioglioma, one had pediatric-type diffuse low-grade glioma, and one had atypical teratoid rhabdoid tumor (ATRT). The outcomes of low-grade tumors were favorable, especially for those who underwent resection, and those of high-grade tumors were poor regardless of the extent of resection.

Conclusion: Our review shows that surgical resection of thalamic tumors can be done safely and offers favorable outcomes for patients with low-grade tumors, even without adjuvant therapy. Our study provides further evidence for thalamic tumors operability and safe resection.

## Introduction

Thalamic tumors represent approximately 1-5% of pediatric brain tumors [1]. Because of the essential functions of the thalamus and surrounding structures, less aggressive approaches such as biopsy and adjuvant therapy are generally preferred due to the high risk of morbidity and mortality associated with radical surgical resection [2,3]. Advancements in neuroimaging, intraoperative neurophysiological monitoring, and neuronavigational technology have improved the safety of surgical resection of thalamic tumors, with reasonable morbidity [4-8]. Here, we review the surgical outcomes of 10 pediatric patients diagnosed with thalamic tumors. We demonstrate the rule of high-definition fiber tractography and the utilization of the fiber tracking technique to achieve safe surgical resection.

## Materials and methods

We reviewed the medical records of 10 pediatric patients diagnosed with thalamic tumors at the Department of Pediatric Neurosurgery National Neuroscience Institute/King Fahad Medical City. All related medical data, including history and physical examination, imaging findings, operative reports, histopathology, adjuvant therapy, and follow-up data, were retrieved. MRI of the brain was performed in all patients to determine the tumor location and relation to the surrounding structures. Diffusion tensor tractography (DTI) was done in all patients preoperatively. All images were reviewed and reported by neuroradiologists. Neuronavigation was performed in all patients, and the fiber tracking technique was used and illustrated in one patient (case illustration). Intraoperative monitoring IOM was used in all patients except those who underwent biopsy. Based on intraoperative and postoperative MRI, the extent of resection was categorized into gross total resection (GTR, 100% removal), subtotal resection (STR, 90-99% removal), and partial resection (PR, <90% removal). Neurological evaluations including mental status assessment and power examinations were performed in all patients, and accordingly, the patients were divided into two groups: stable, compared with the preoperative evaluation, and worsened.

## Results

Clinical characteristics

Ten patients were reviewed and included in the study. Their ages ranged from three to 13 years. The male-to-female ratio was 3/7. Hemiparesis was the most common symptom (n=8), followed by increased intracranial pressure (n=5). Headache and dizziness were the presenting symptoms of two patients, and they had no neurological deficits.

Radiological features

All patients had a unilateral thalamic tumor - three had a right-sided thalamic tumor and seven had a left-sided thalamic tumor. Three had thalamopeduncular tumors, three had thalamic tumors with intraventricular extension, two had thalamic tumors with extension through the tentorium, one had a mesial thalamic tumor, and one had a thalamotemporal tumor. DTIs were performed in all patients to evaluate the relationship between the tumor and the basal ganglia and internal capsule and to choose the safest surgical route.

Surgical intervention and extent of resection

We performed three biopsies and seven tumor resections. Three cases had GTRs, two had STRs, and two had PRs. Surgical routes were selected based on the evaluation of DTI images preoperatively. Four tumor resections were performed via transcortical routes and three via transcortical-transventricular routes. Two stereotactic biopsies and one endoscopic biopsy were performed.

Neurological status after surgery and histopathological findings

Among tumor resection cases, only one patient worsened after surgery, but the patient returned to the baseline neurological status after one month. Two cases remained stable. Four cases improved after surgery. The histopathological diagnoses of the 10 patients were as follows: five patients had pilocytic astrocytoma, two had diffuse pediatric-type high-grade glioma, one had ganglioglioma, one had pediatic-type diffuse low-grade glioma, and one had atypical teratoid rhabdoid tumor (ATRT) [9].

Adjuvant therapy and follow-up

All high-grade tumor cases had chemotherapy and radiotherapy following surgery. Among low-grade tumor cases, only one patient had chemotherapy after surgery. The mean follow-up time of the low-grade tumor cases was 27.5 months, and that of the high-grade tumor cases was 6.2 months.

Outcome

Among low-grade tumor cases, no death event was reported during the whole follow-up period (six to 96 months). There was no tumor recurrence or residual progression, but there was one case of tumor regression after chemotherapy. All high-grade tumor cases had tumor progression, and two death events were reported (Table 1).

**Table 1:** Patients characteristics and follow-up GTR: gross total resections; STR: subtotal resections; ATRT: atypical teratoid rhabdoid tumor; M: male; F: female

Case no.	Age (years)	Gender	Pre-op power	Tumor location	Surgical approach	Grade of resection	Histopathology	Adjuvant therapy	Recurrence	Follow-up time (months)	Postoperative power
1	11	M	2/5	Left thalamopeduncular	Left transcortical-transventricular	PR	Pilocytic astrocytoma	No	Stable	6	4/5
2	8	F	4/5	Left thalamopeduncular	Left stereotactic biopsy	Biopsy	Pilocytic astrocytoma	Chemotherapy	Regression	10	4/5
3	12	F	4/5	Left thalamus with extension to tentorium	Left transcortical	PR	Diffuse pediatric-type high-grade glioma	Chemo/radiation	Progression	5	4/5
4	11	M	1/5	Left thalamus with intraventricular extension	Left transcortical-transventricular	GTR	Pilocytic astrocytoma	No	No	12	3/5
5	5	F	5/5	Right thalamus with intraventricular extension	Right frontal endoscopic biopsy	Biopsy	Ganglioglioma	No	Stable	5	5/5
6	13	F	5/5	Left medial thalamus	Left stereotactic biopsy	Biopsy	Pediatric-type diffuse low-grade glioma	No	Unknown	Lost	5/5
7	7	F	3/5	Right thalamopeduncular	Right transcortical	GTR	Pilocytic astrocytoma	No	No	36	4/5
8	3	M	1/5	Right thalamus with intraventricular extension	Right transcortical-transventricular	STR	ATRT	Chemo/radiation	Progression	5	3/5
9	9	F	3/5	Left thalamic extension through the tentorium	Left transcortical	STR	Diffuse pediatric-type high-grade glioma	Chemo/radiation	Progression	9	3/5
10	8	F	4/5	Left thalamus temporal	Left transcortical	GTR	Pilocytic astrocytoma	No	No	96	2/5

## Discussion

Thalamic tumors are considered inoperable tumors, and radical surgical resection is not recommended because of the associated morbidities and poor outcomes [10]. Biopsy and, to some extent, PR followed by adjuvant therapy are the recommended treatment [10,11]. GTR and STR have been found to improve the survival of patients with thalamic tumors [12,13].

In our study, we found that GTR and STR were associated with a high survival rate and low recurrence rate and residual progression, particularly in low-grade tumor cases, even without adjuvant therapy. In high-grade tumor cases, the outcome was considered to be poor because two patients who underwent STR passed away and one patient who underwent PR had progression during chemotherapy and radiation therapy. We were able to perform a radical resection with one transient morbidity and no death events related to the surgery. Radical surgical resection (GTR and STR) was achieved in 50% of our cases, which is comparable to the results of other series [3,12,14]. Although we had only 10 cases in our study and the condition of one patient (10%) worsened temporarily, the neurological outcomes of our study were slightly better than those of other studies [15,16]. Our review seems to provide further evidence supporting radical resection of thalamic tumors in the pediatric population.

Preoperative planning, employment of DTI, neuronavigation, and intraoperative neurophysiologic monitoring are the cornerstones of surgical safety in addressing thalamic tumors. Utilization of DTI to select the surgical route in thalamic tumors has been discussed in multiple studies [8,15-18]. Selection of the safest route depends on the identification of the normal thalamus, internal capsule, and tumor extension into adjacent structures [18].To achieve maximal safe resection, avoiding these structures, particularly the corticospinal tract in the posterior limb of internal capsule (PLIC), is essential. In our center, we perform DTI in all patients diagnosed with thalamic tumor. We discuss all cases with our neuronavigation consultant to formulate the plan and 3D model of the tumor, thalamus, internal capsule, and surrounding vital structures, and then, the surgical route selected accordingly. We do not prefer one surgical route over another, but the route that avoids transecting the PLIC and normal thalamus, namely, the transcortical or transcortical-transventricular route in our study, is the route of choice.

Currently, we employed high-definition fiber tracking (HDFT) in our cases, and we present an illustrative case. We found it to be a very useful tool that provides us with clear and precise visualization during surgical planning and intraoperatively. HDFT is used to identify the entry point, trajectory and corticospinal tract (CST), and interface between the thalamus and tumor. There is only one case series in the current literature that utilized HDFT in the surgical treatment of thalamic tumors in a pediatric population [19]. Celtikci et al. reported a series of three pediatric patients with thalamopeduncular tumors who underwent surgical resection [19]. They found that the CST was displaced anteromedially in two patients and anteriorly in one patient and not infiltrated by the tumor. In our patient, the CST was displaced anterolaterally and at the level of the left cerebral peduncle deviation posterolaterally and was not infiltrated by the tumor. Further studies are needed to examine the use of HDFT in the surgical treatment of thalamic tumors and evaluate its impact on the extent of tumor resection and functional outcome.

Illustrative case

**Figure 1:**
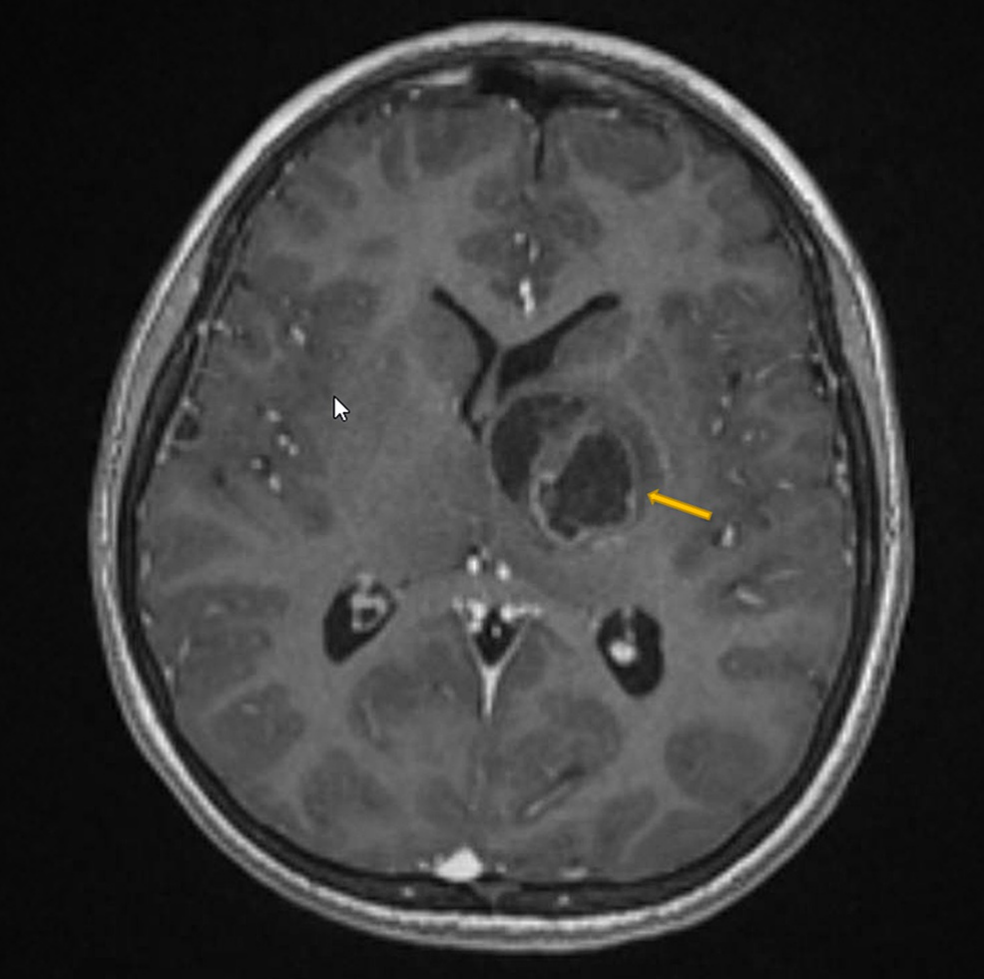
Preoperative axial T1-weighted MRI with contrast The image shows a left thalamic lesion, a heterogeneous mass with internal necrosis, and hemorrhagic components centered within the left thalamus with involvement of the left cerebral peduncle and basis of the midbrain, suggestive of high-grade glioma.

An 11-year-old boy presented with a three-week history of progressive weakness. His examination revealed right-sided hemiparesis with motor power 2/5. MRI demonstrated a heterogeneous mass with internal necrosis and hemorrhagic components centered within the left thalamus with involvement of the left cerebral peduncle and basis of the midbrain suggestive of high-grade glioma (Figure 1). Diffusion tensor images demonstrate a preserved cortical spinal tract and a mass effect on the left internal capsule fibers that deviated laterally. At the level of the left cerebral peduncle, pyramidal tracts are compressed and deviated posterolaterally without evidence of invasion (Figure 1).

**Figure 2:**
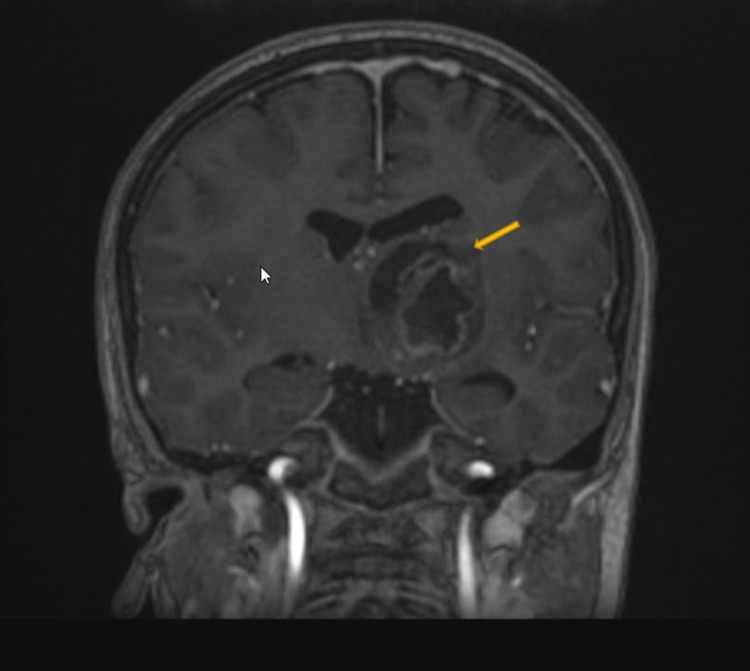
Preoperative coronal T1-weighted MRI and DTI with contrast The image shows left thalamic mass with heterogenous enhancement and mass effect on the third ventricle. DTI: diffusion tensor imaging

**Figure 3:**
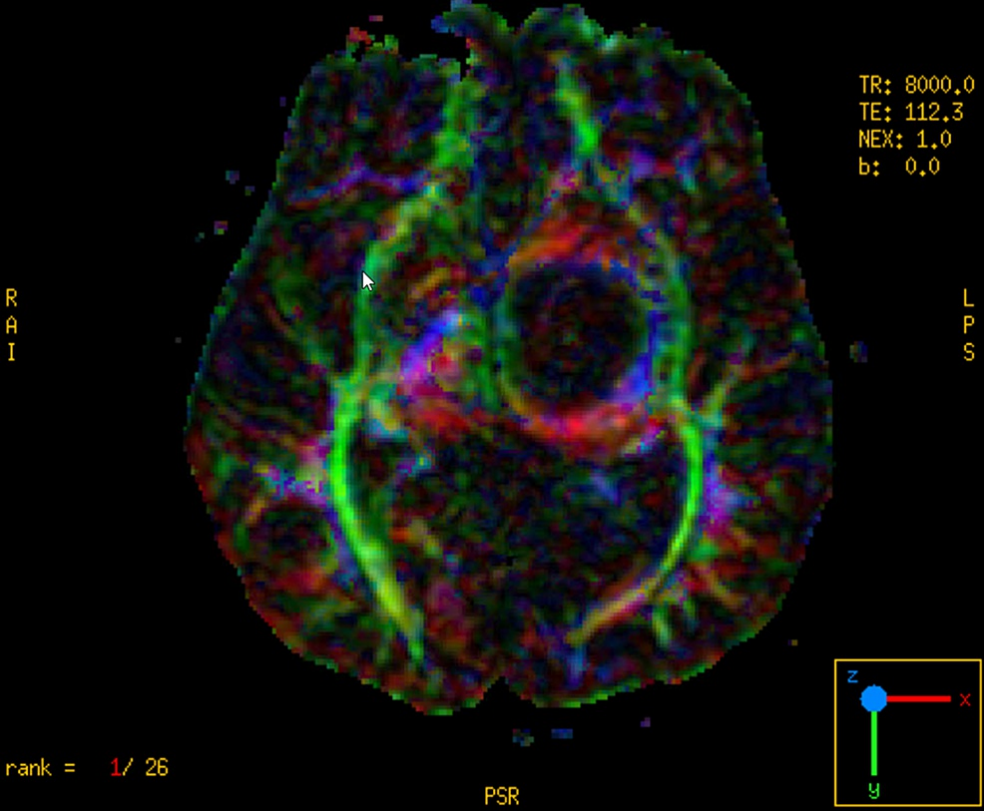
Preoperative axial MRI and DTI image shows deviation of PLIC posterolaterally. DTI: diffusion tensor imaging; PLIC:

**Figure 4:**
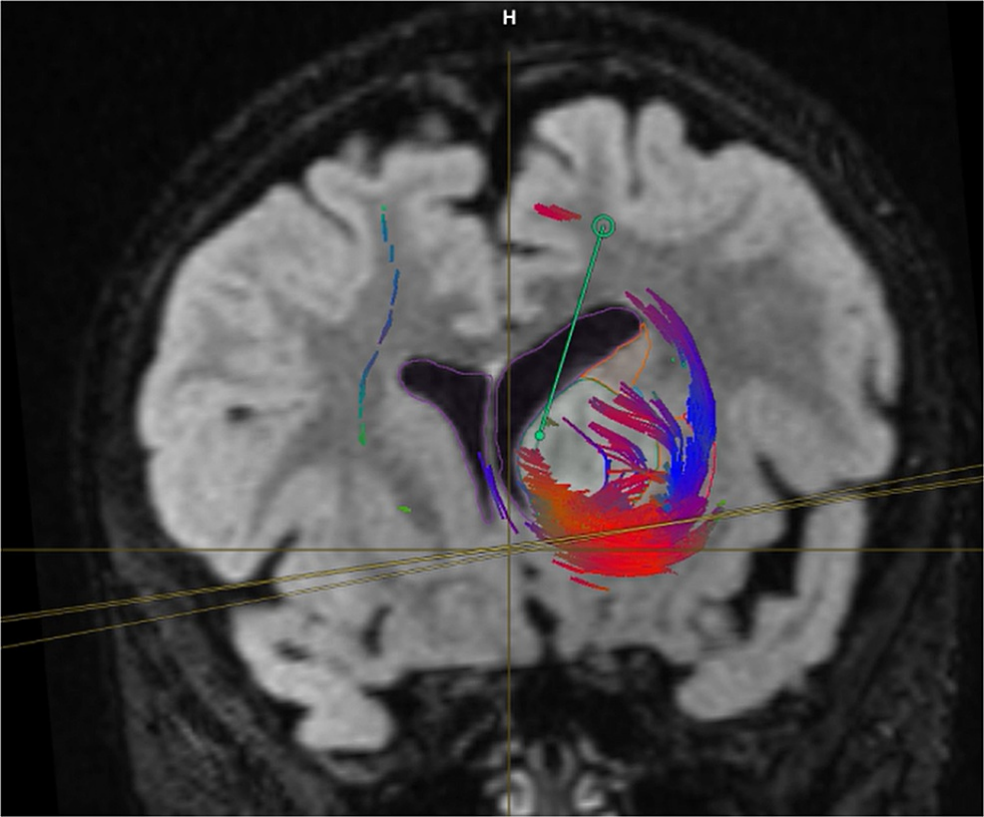
Coronal MRI integrated with high-resolution fibers Illustrating the surgical plan via high-definition tractography and fiber tracking The image shows the entry point via the transcortical-transventricular route directly to the tumor where there are no critical fibers in the way.

**Figure 5:**
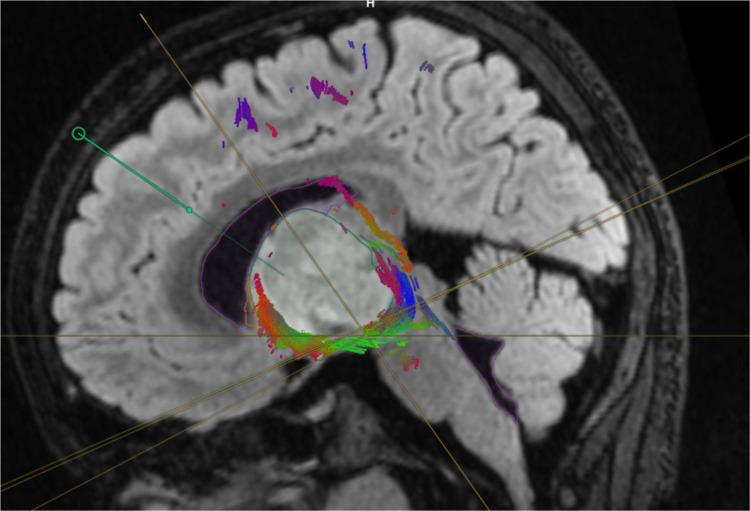
Sagittal view Illustration of surgical plan via high-definition tractography and fiber tracking The image demonstrates the tumor and fiber interface and the surgical entry point and trajectory.

**Figure 6:**
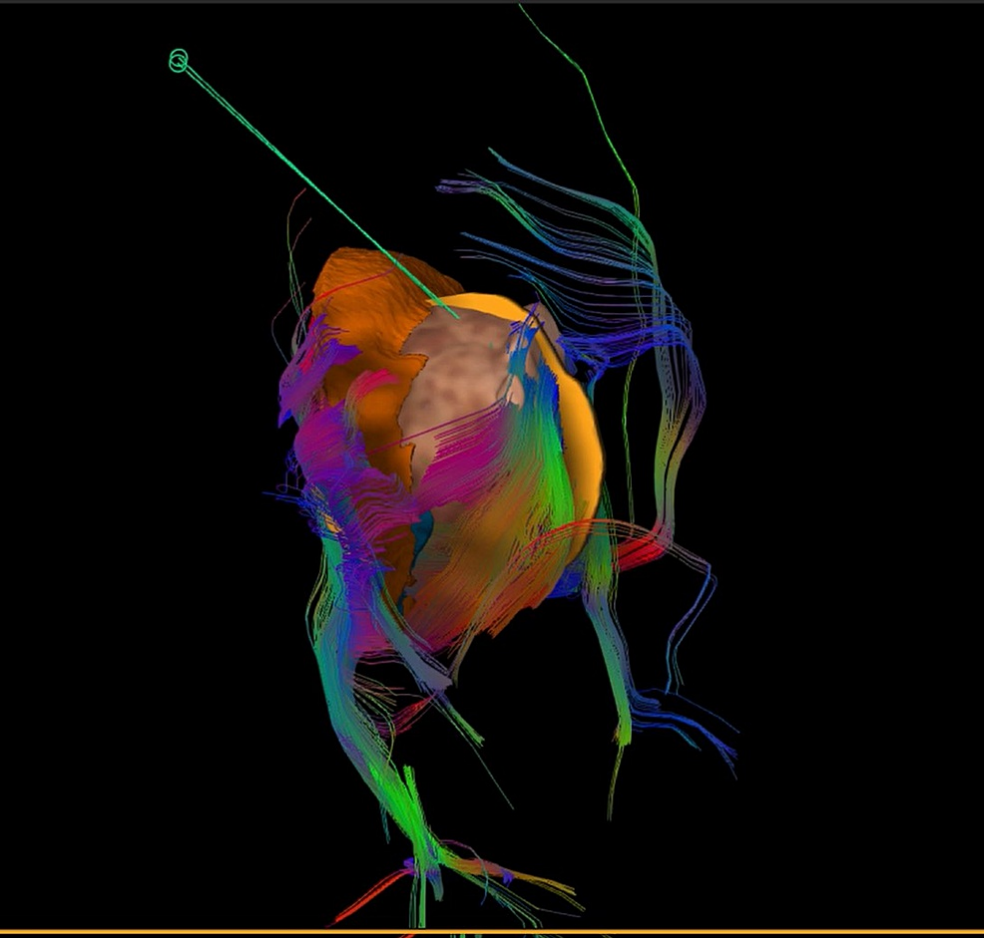
Three-dimensional Illustration of surgical plan via high-definition tractography and fiber tracking Three-dimensional reconstruction of the thalamus, tumor, fibers, and trajectory after subtraction of brain tissue and ventricle.

**Figure 7:**
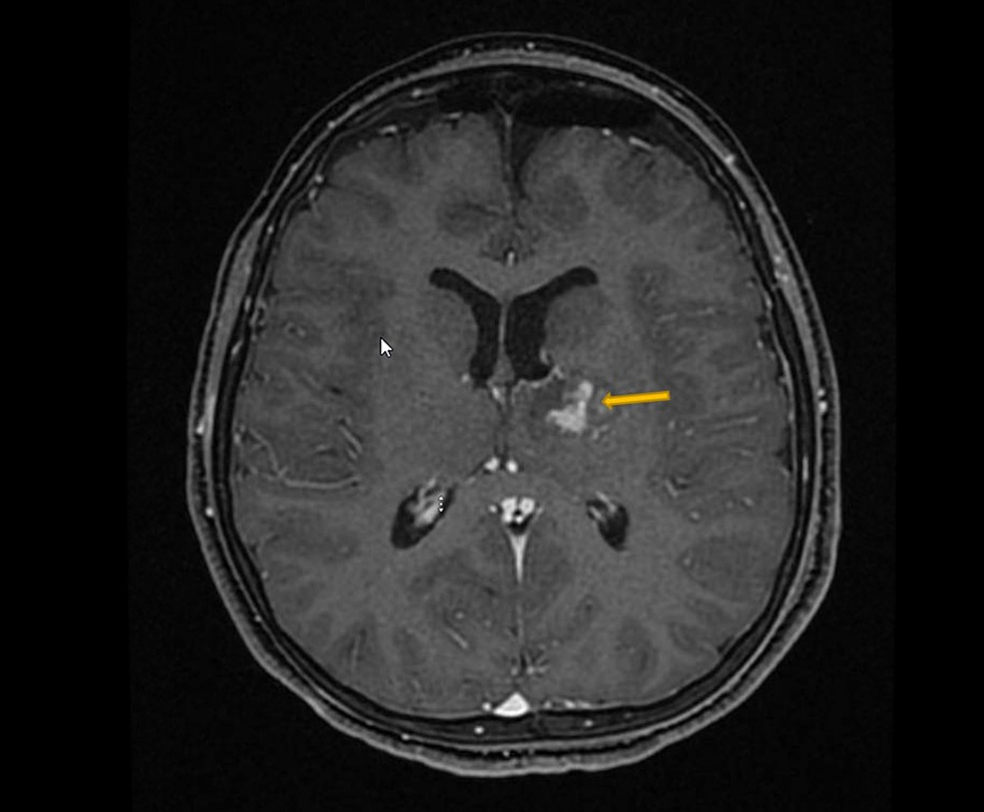
Postoperative T1-weighted MRI with contrast The image shows the residual tumor and resolution of the mass effect on the third ventricle.

Neuronavigation integrated with DTI and fiber tracking was performed (Figure 2). The patient underwent the left frontal transcortical-transventricular approach under IOM. Analysis of a frozen specimen by pathology showed low-grade glioma, so we decided to perform tumor resection. Immediately after surgery, his power improved to 4/5, and his postoperative MRI showed a 70% reduction in tumor size. His postoperative DTI revealed preservation of the left CST (Figures 3-7).

## Conclusions

Our review shows that surgical resection of thalamic tumors can be done safely and offers a favorable outcome for low-grade tumors, even without adjuvant therapy. High-definition fiber tractography and utilization of the fiber tracking technique might be considered to maximize the extent of resection safely. The ability to identify low-grade tumors based on images needs further studies and will help neurosurgeons in decision making and surgical planning.
